# Pediatric Trapped Fourth Ventricle: The Need for Objective Surgical‐Selection Criteria and Long‐Term Surveillance After Open Fenestration

**DOI:** 10.1002/ccr3.73389

**Published:** 2026-08-24

**Authors:** Muhammad Abdullah Awan, Hammad‐ud‐din Raza

**Affiliations:** ^1^ Wah Medical College National University of Medical Sciences Rawalpindi Pakistan

**Keywords:** isolated fourth ventricle, neurosurgical follow‐up, open fenestration, pediatric hydrocephalus, trapped fourth ventricle, ventriculoperitoneal shunt

## Abstract

Open fenestration can be an effective treatment for pediatric trapped fourth ventricle, but case reports should move beyond successful short‐term decompression. Future reports should include objective surgical‐selection criteria, intraoperative confirmation of adhesions, standardized imaging metrics, and long‐term surveillance end points to clarify durability and recurrence risk.

Dear Editor,

We read with great interest the case report and literature review by Hamza et al. [1], titled “Trapped Fourth Ventricle in a Pediatric Patient With a History of Post‐Traumatic Hydrocephalus and Ventriculoperitoneal Shunt,” published in *Clinical Case Reports*. The authors describe a 7‐year‐old boy with prior ventriculoperitoneal shunting for post‐traumatic hydrocephalus who presented with progressive drowsiness, gait disturbance, behavioral change, and episodic loss of consciousness. Initial CT raised suspicion of a posterior fossa tumor, whereas MRI correctly demonstrated a markedly dilated fourth ventricle with brainstem and cerebellar compression, establishing the diagnosis of trapped fourth ventricle (TFV) [1].

The report is clinically valuable because it reminds clinicians that TFV may mimic posterior fossa tumors and that MRI is essential for accurate diagnosis in shunted children with new posterior fossa or brainstem symptoms. The authors also provide an important management example: open posterior fenestration with microsurgical arachnoid adhesiolysis, followed by postoperative reduction in fourth ventricular size and complete clinical improvement at 1‐month follow‐up [1]. However, one aspect deserves stronger emphasis: In pediatric TFV, the key question is not only whether decompression succeeds in the short term, but whether the surgical decision pathway is reproducible and durable.

The authors selected open posterior fenestration rather than an endoscopic approach because dense arachnoid adhesions were clinically suspected in view of the patient's history of trauma and previous surgery [1]. This reasoning is plausible and clinically defensible. Nevertheless, future reports should clarify the objective criteria used to choose open fenestration over endoscopic aqueductoplasty, fourth ventricular shunting, stenting, or combined shunt strategies. Useful preoperative variables may include the number of previous shunt procedures, history of infection or hemorrhage, presence of posterior fossa surgery, aqueductal patency, foraminal obstruction, cisterna magna scarring, supratentorial ventricular size, and evidence of fourth ventricular outlet obstruction.

A related issue is intraoperative confirmation. The article states that open surgery was chosen to allow direct visualization and lysis of potential adhesions [1]. Future reports should specify whether adhesions were actually encountered, where they were located, how dense they were, whether the foramina of Magendie or Luschka were obstructed, and whether a durable communication with the cisterna magna was confirmed intraoperatively. These details would convert a reasonable operative choice into a reproducible surgical‐selection framework. Without such information, readers cannot easily determine whether the same patient would have been suitable for endoscopic treatment in a center with different expertise or equipment.

Imaging reporting could also be standardized. The preoperative MRI and postoperative CT are central to the case, but the report would be strengthened by quantitative or semiquantitative imaging end points. These may include fourth ventricle dimensions, degree of brainstem compression, status of the aqueduct, size of the lateral and third ventricles, presence of slit ventricles, position and function of the existing VP shunt, and interval change after surgery. Where available, cine MRI or other CSF‐flow assessment could help distinguish static ventricular enlargement from functionally obstructed fourth ventricular outflow. Even when advanced imaging is unavailable, basic measurements would improve comparability across reports.

The language of treatment success should also be interpreted cautiously. The patient's recovery at 1 month is encouraging, and the day‐10 CT demonstrated a reduction in fourth ventricular size [1]. However, TFV may recur because of readhesion, restenosis, shunt malfunction, altered supratentorial CSF dynamics, or progressive compartmentalization. Therefore, short‐term symptom resolution should not be equated with durable treatment success unless longer follow‐up is available. The authors appropriately acknowledge the need for continued surveillance, and future case reports should make this surveillance plan explicit.

A minimum follow‐up dataset for pediatric TFV could include neurological status, gait and coordination assessment, cranial nerve examination, swallowing and speech evaluation when relevant, school or developmental progress, parental warning signs, shunt‐function assessment, repeat imaging intervals, and criteria for reintervention. Such data are especially important in children because brainstem compression and hydrocephalus‐related injury may affect neurodevelopment, behavior, and functional recovery beyond the immediate postoperative period.

The literature table included by Hamza et al. [1] is useful because it summarizes shunt‐based, endoscopic, open microsurgical, and mixed approaches. Its broader lesson, however, is that outcome reporting remains heterogeneous. Future TFV literature would benefit from standardized end points: complete versus partial symptom resolution, fourth ventricular size reduction, recurrence, need for reoperation, shunt revision, stent migration, cranial nerve deficits, infection, and length of follow‐up. This would allow clinicians to compare open fenestration, endoscopic aqueductoplasty, and fourth ventricular shunting more reliably rather than relying on isolated successful outcomes.

Importantly, these comments do not diminish the educational contribution of the case. Hamza et al. [1] present a valuable example of accurate MRI‐based diagnosis and successful operative management of a rare pediatric shunt complication. Rather, we suggest that future reports should frame TFV management as a complete pathway: suspicion, imaging confirmation, selection of operative strategy, intraoperative verification of the presumed mechanism, and long‐term monitoring for recurrence. This approach would improve the translational value of individual cases and help define practical standards in settings with variable neurosurgical resources.

This case effectively highlights that TFV should be suspected in shunted children with new neurological deficits and posterior fossa findings. However, future pediatric TFV case reports should include objective criteria for selecting open versus endoscopic or shunt‐based treatment, intraoperative confirmation of adhesions or obstruction, standardized imaging measurements, and long‐term surveillance end points. These additions would shift the literature from successful short‐term decompression toward reproducible, durable, and clinically comparable management strategies.

## Author Contributions

**Muhammad Abdullah Awan:** conceptualization, formal analysis, writing – original draft, writing – review and editing, data curation, project administration. **Hammad‐ud‐din Raza:** conceptualization, formal analysis, writing – original draft, writing – review and editing.

## Funding

The authors have nothing to report.

## Ethics Statement

The authors have nothing to report.

## Consent

The authors have nothing to report.

## Conflicts of Interest

The authors declare no conflicts of interest.

## Data Availability

Data sharing not applicable to this article as no datasets were generated or analysed during the current study.
